# Evolution of Karyotypes in Chameleons

**DOI:** 10.3390/genes8120382

**Published:** 2017-12-12

**Authors:** Michail Rovatsos, Marie Altmanová, Martina Johnson Pokorná, Petr Velenský, Antonio Sánchez Baca, Lukáš Kratochvíl

**Affiliations:** 1 Department of Ecology, Faculty of Science, Charles University, 12844 Prague, Czech Republic; michail.rovatsos@natur.cuni.cz (M.R.); altmanova.m@gmail.com (M.A.); martina.pokorna@natur.cuni.cz (M.J.P.); 2 Institute of Animal Physiology and Genetics, The Czech Academy of Sciences, 27721 Liběchov, Czech Republic; 3 Prague Zoological Garden, 17100 Prague, Czech Republic; velensky@zoopraha.cz; 4 Department of Experimental Biology, Faculty of Experimental Sciences, University of Jaén, 23071 Jaén, Spain; abaca@ujaen.es

**Keywords:** karyotype evolution, ITS, rDNA, sex chromosomes, ancestral chromosome number, Chamaeleonidae

## Abstract

The reconstruction of the evolutionary dynamics of karyotypes and sex determining systems in squamate reptiles is precluded by the lack of data in many groups including most chameleons (Squamata: Acrodonta: Chamaeleonidae). We performed cytogenetic analysis in 16 species of chameleons from 8 genera covering the phylogenetic diversity of the family and also phylogenetic reconstruction of karyotype evolution in this group. In comparison to other squamates, chameleons demonstrate rather variable karyotypes, differing in chromosome number, morphology and presence of interstitial telomeric signal (ITS). On the other hand, the location of rDNA is quite conserved among chameleon species. Phylogenetic analysis combining our new results and previously published data tentatively suggests that the ancestral chromosome number for chameleons is 2n = 36, which is the same as assumed for other lineages of the clade Iguania, i.e., agamids and iguanas. In general, we observed a tendency for the reduction of chromosome number during the evolution of chameleons, however, in *Rieppeleon brevicaudatus*, we uncovered a chromosome number of 2n = 62, very unusual among squamates, originating from a number of chromosome splits. Despite the presence of the highly differentiated ZZ/ZW sex chromosomes in the genus *Furcifer*, we did not detect any unequivocal sexual differences in the karyotypes of any other studied species of chameleons tested using differential staining and comparative genomic hybridization, suggesting that sex chromosomes in most chameleons are only poorly differentiated.

## 1. Introduction

Chameleons (family Chamaeleonidae) are morphologically unique lizards with several exceptional characteristics such as projectable tongue, independently movable eyes, prehensile feet, and the ability in many species to change the colour of their skin. They are found in Africa, Madagascar, Southern Europe and Southern Asia. Chameleons are a sister group to dragon lizards (family Agamidae) and have a relatively recent origin with the basal split dated to approximately 65 million years ago [1]. Despite the general interest in this group, chameleons are relatively poorly studied cytogenetically and karyotypes are known for only 59 out of about 200 extant species [2,3]. The chromosome number is rather variable, ranging from 2n = 20 to 2n = 36 [3]. 

The karyotypes have mostly been studied using classical cytogenetic techniques. Little is also known about sex determination in chameleons. The observation of equal sex ratio across several incubation temperatures suggests that chameleons possess genotypic sex determination. The earlier reports on environmental sex determination in this group are not conclusive [4,5,6], however, sex chromosomes within this clade were unequivocally detected only very recently. In our previous study, we used molecular cytogenetic methods to uncover highly differentiated sex chromosomes in two species of the genus *Furcifer* [7]. The Malagasy giant chameleon (*Furcifer oustaleti*) (chromosome number 2n = 22) possesses heteromorphic Z and W sex chromosomes, whereas the panther chameleon (*Furcifer pardalis*) (2n = 22 in males, 2n = 21 in females) exhibits among vertebrates [8,9] the rather rare Z_1_Z_1_Z_2_Z_2_/Z_1_Z_2_W system of multiple sex chromosomes, which most likely evolved via W-autosome fusion. However, representatives of other genera have not yet been studied by molecular cytogenetics and their sex chromosomes remain unknown.

Here we reviewed all available data regarding diploid chromosome number and other cytogenetic characteristics for the family Chamaeleonidae and combined our findings with our new results. We performed phylogenetic analysis in other species to uncover evolutionary dynamics and trends of karyotype evolution within this group. We cytogenetically examined 16 species of the family from eight genera with the use of molecular cytogenetic methods. As several squamate lineages show stability in differentiated sex chromosomes [10,11,12,13,14,15,16,17], we tested whether differentiated sex chromosomes recently described in the genus *Furcifer* [7] are also present in other chameleons.

## 2. Materials and Methods

### 2.1. Studied Material

We examined 52 individuals originating from the pet trade and zoological gardens (Zoo Plzeň, Zoopark Zájezd) and the CITES centre for confiscated animals in Prague Zoo (Table S1). The processing of the biological material was carried out under the supervision and with the approval of the Ethics Committee of the Faculty of Science, Charles University in Prague followed by the Committee for Animal Welfare of the Ministry of Agriculture of the Czech Republic (No. 29555/2006-30).

### 2.2. DNA Barcoding

In order to properly identify our specimens and to characterize them for future studies, we applied DNA barcoding [18]. Genomic DNA was extracted using a DNeasy Blood and Tissue Kit (Qiagen, Valencia, CA, USA), and the 5′ fragment of the mitochondrial cytochrome oxidase I COI gene [18,19] was amplified by PCR, using either the reptile-specific primers RepCOI-F and RepCOI-R [18] or the universal primers LCO1490 and HCO2198 [19]. The PCR reaction and cycling conditions are described in [20]. The PCR products were sequenced bidirectionally by Macrogen (Seoul, Korea). The COI sequences were aligned using CLUSTALW [21] included in BioEdit v5.0.9 [22] and subsequently analysed in DNAsp v5.10.1 [23]. Genetic distances among haplotypes were calculated using the Kimura 2-parameter model in MEGA v6.0.5 [24]. A BLAST search was performed to compare our sequences with those from previous studies.

### 2.3. Chromosome Preparation and Staining

Metaphase chromosome spreads were prepared from whole blood cell cultures, following the protocol described in [25]. Briefly, a small amount (approx. 40 μL) of peripheral blood was cultured for a week at 30 °C in Dulbecco’s Modified Eagle’s Medium (Sigma-Aldrich, St. Louis, MO, USA), enriched with 10% fetal bovine serum (Thermo Fisher Scientific, Waltham, MA, USA), 0.5% penicillin/streptomycin solution (Thermo Fisher Scientific, Waltham, MA, USA), 1% l-glutamine (Sigma-Aldrich, St. Louis, MO, USA), 3% phytohaemagglutinin (Thermo Fisher Scientific, Waltham, MA, USA) and 1% lipopolysaccharide (Sigma-Aldrich, St. Louis, MO, USA). Chromosome preparations were made following standard procedures including a colcemid treatment for 3.5 h, hypotonization with 0.563% KCl for 30 min and fixation in 3:1 methanol: acetic acid. Chromosomal preparations from all specimens were stained with conventional Giemsa solution. C-banding was performed as described by [26] with slight modifications, i.e., the slides were aged at 65 °C for 1 h, soaked in 0.2N HCl for 20 min, then in 5% Ba(OH)_2_ solution for 4.5 min at 45 °C and then rinsed in 0.2N HCl. Finally, the slides were soaked in 2× saline-sodium citrate (SSC) buffer for 1 h at 60 °C, rinsed in distilled water, and stained with 4′,6-diamidino-2-phenylindole (DAPI).

### 2.4. Comparative Genomic Hybridization 

Male and female genomic DNA were labelled with biotin- deoxyuridine triphosphate (dUTP) and digoxigenin-dUTP, respectively, using a Nick Translation Kit (Abbott Laboratories, Lake Bluff, IL, USA). From each sample, 1 μg of male and 1 μg of female labelled genomic DNA was co-precipitated overnight with 5 μL salmon sperm DNA (10 mg/mL, Sigma), 10 μL of 3M sodium acetate and 2.5× volume of ethanol. After precipitation, the dry pellets were resuspended in 22 μL hybridization buffer (50% formamide, 2× SSC, 10% SDS, 10% dextran sulfate, 1× Denhardt’s buffer, pH 7), denatured at 75 °C for 10 min and then chilled on ice for 10 min prior to hybridization. At the same time, the metaphase slides were treated with RNase and pepsin, fixed with 4% formaldehyde, dehydrated through a 70, 85 and 100% ethanol series, denatured in 70% formamide/2× SSC at 75 °C for 3 min and dehydrated again. For the next step, 11 μL of the probe (concentration approx. 500 ng of labeled DNA) was applied on the slide per drop of chromosomal suspension and incubated at 37 °C for 48 h. Post-hybridization washes were performed in 50% formamide/2× SSC at 42 °C and in 2× SSC. Each slide was incubated with 100 μL of 4× SSC/5% blocking reagent (Roche, Basel, Switzerland) at 37 °C for 30 min and then with 100 μL detection solution 4× SSC/5% blocking reagent including 2 μL of avidin-FITC (Vector Laboratories, Burlingame, CA, USA) and 10 μL of anti-digoxigenin-rhodamine (Roche, Basel, Switzerland) at 37 °C for 30 min. The slides were subsequently washed in 4× SSC/0.05% Tween 20, dehydrated through an ethanol series and air dried. Finally, the slides were mounted with Fluoroshield antifade medium containing DAPI (Sigma-Aldrich, St. Louis, MO, USA). For our detailed protocol see [27].

### 2.5. Fluorescence In Situ hybridization with Telomeric Probe and rRNA Gene

The topology on the karyotype of the telomeric motif (TTAGGG)n and the rRNA genes within the genomes were analysed by fluorescence *in situ* hybridization (FISH). The telomeric probe was produced and labelled with biotin in a single PCR reaction using the primers (TTAGGG)5 and (CCCTAA)5, without a DNA template [28]. The rRNA gene probe was prepared from a plasmid (pDm r.a 51#1) with a 11.5 kb insert, encoding the 18S and 28S ribosomal units of *Drosophila melanogaster*. The probe was labelled with biotin-dUTP, using a Nick Translation Kit (Abbott Laboratories, Lake Bluff, IL, USA).

In both cases, the probe was ethanol-precipitated together with salmon sperm DNA, resuspended in hybridization buffer (50% formamide/2× SSC), then denatured at 75 °C for 6 min and chilled on ice for 10 min prior to hybridization. The chromosomal preparations were treated as in comparative genomic hybridization (CGH). Hybridization was performed at 37 °C overnight, followed by post-hybridization washes with 50% formamide/2× SSC at 42 °C for 5 min (3 times) and 2× SSC for 5 min (3 times). The slides were incubated in 100 μL of 4× SSC/5% blocking reagent (Roche, Basel, Switzerland) at 37 °C for 45 min and then with 100 μL of 4× SSC/5% blocking reagent containing avidin-FITC (Vector Laboratories). The fluorescence signal was enhanced and detected using a modified avidin-FITC/biotinylated anti-avidin system (Vector Laboratories, Burlingame, CA, USA), according to [27]. Finally, the slides were mounted with Vectashield DAPI anti-fade medium (Vector Laboratories, Burlingame, CA, USA).

### 2.6. Microdissection and Chromosome Painting

In *Trioceros johnstoni* we observed a heteromorphic pair of chromosomes in the female metaphases. We tested whether they have female-specific sequence content using microdissection and subsequent hybridization of the probe to metaphases of the same species. Microdissection was performed using an inverted microscope (Zeiss Axiovert S100, Oberkochen, Germany) with a sterile glass needle attached to a mechanical micromanipulator (Eppendorf TransferMan NK2, Hamburg, Germany). A total of 15-20 microdissected chromosomes were used as templates for DNA amplification by degenerate oligonucleotide-primed PCR (DOP-PCR) [29]. Primary DOP-PCR product was used as a template in a secondary DOP-PCR to incorporate Spectrum-Orange-dUTP (Roche, Basel, Switzerland). Two probes were prepared from each of the 7th pair chromosomes in the female *T. johnstoni* (heteromorphic chromosomes) and a single probe also from the 7th pair in the male (homomorphic chromosomes). Chromosome painting was performed according to the protocol of [30].

### 2.7. Microscopy and Image Analyses

Images were captured using a Provis AX70 (Olympus, Tokyo, Japan) fluorescence microscope equipped with a DP30BW digital camera (Olympus Tokyo, Japan). The karyotype was arranged using Ikaros karyotyping software (Metasystems, Altlussheim, Germany). DP manager imaging software (Olympus, Tokyo, Japan) was used to capture greyscale images and to superimpose the source images with colours to visualize the results of the FISH.

### 2.8. Phylogenetic Analyses

Our new data were combined with the published data on karyotypes in chameleons and used in the phylogenetic analyses. The reconstruction of the ancestral chromosome number was performed in Mesquite [31] using the maximum parsimony approach using the phylogenetic hypotheses proposed by [1,32]. These two recent phylogenies differ rather extensively in the position of several genera and even in the support for monophyly of the genus *Calumma*. We excluded *Furcifer rhinoceratus* with known chromosome number (2n = 22) [33,34] from the tree by Pyron et al. [32] as this species is missing there. Also, we did not include *Furcifer lateralis* (reported to have 2n = 24 in [35]) as this group underwent notable taxonomic changes since that time. However, its inclusion would not affect the reconstruction as the chromosome number of this species is within the range of chromosome numbers in the genus.

## 3. Results

### 3.1. DNA Barcoding

A fragment of the mitochondrial gene COI was successfully amplified by PCR in 15 species. In *Kinyongia xenorhina* we lacked enough material for DNA isolation. We revealed 19 unique haplotypes. All haplotypes were deposited in GenBank and a BLAST search was carried out for each (Table S1). High similarities (more than 98%) for COI sequences present in genome databases were revealed for *B. stumpffi*, *C. brevicorne*, *C. globifer*, *C. malthe*, *C. parsonii*, *C. calyptratus* and *T. melleri*. Comparisons were not possible for *B. thamnobates*, *K. boehmei*, *R. temporalis*, *R. brevicaudatus*, *T. bitaeniatus*, *T. hoehnelii* and *T. johnstoni*, as reference sequences from these species were not identified in the BLAST search. Surprisingly, a single haplotype (derived from six individuals) had only a partial similarity with reference sequences of other *Calumma* species, with *C. crypticum* and *C. brevicorne* being closely related. Considering the large genetic distance of these individuals to the reported references, we chose to refer to them as *C.* cf. *crypticum*.

### 3.2. Cytogenetic Analyses

**Figure 1:**
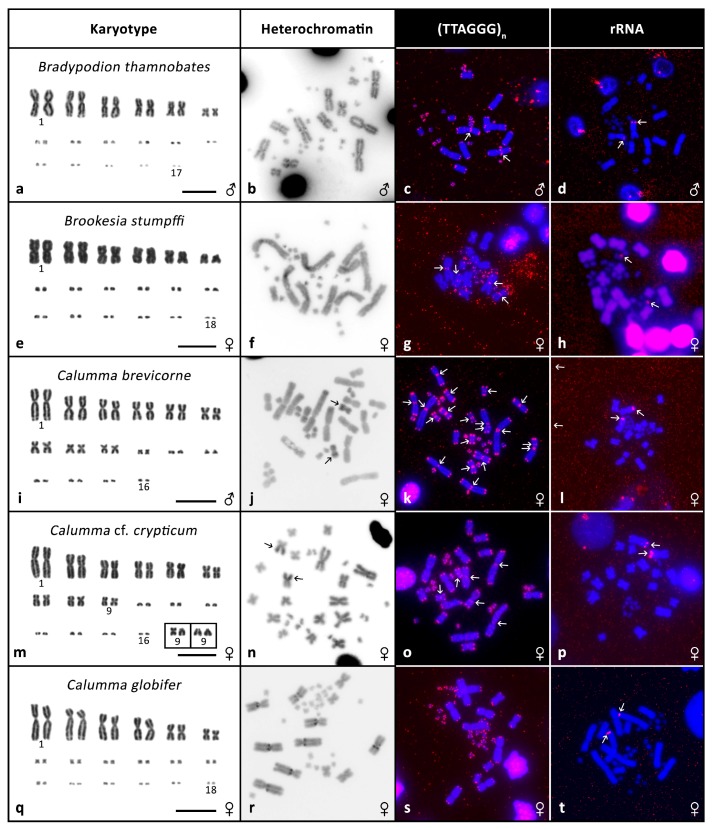
Cytogenetic data. Giemsa stained karyotypes (**a**,**e**,**i**,**m**,**q**), C-banding (**b**,**f**,**j**,**n**,**r**), fluorescence *in situ* hybridization (FISH) with telomeric probe (**c**,**g**,**k**,**o**,**s**) and FISH with rRNA probe (**d**,**h**,**l**,**p**,**t**) in *Bradypodion thamnobates*, *Brookesia stumpffi*, *Calumma brevicorne*, *Calumma* cf. *crypticum*, *Calumma globifer*. Arrows indicate signals.

*Bradypodion thamnobates*: the karyotype is composed of 2n = 34 chromosomes as previously described ([36] reported as unpublished ex [3]). There are 12 biarmed macrochromosomes and 22 microchromosomes, with difficult to identify morphologies (Figure 1a). C-banding revealed the presence of heterochromatin in the pericentromeric regions (Figure 1b) and interstitial telomeric signal (ITS) signals were detected in a pair of macrochromosomes (Figure 1c). FISH with rRNA probe showed a signal in pair 2 (Figure 1d).

*Brookesia stumpffi*: we only had one individual available and due to methodological complications were only able to obtain preliminary data for this species. The karyotype is composed of 2n = 36 with 12 macrochromosomes and 24 microchromosomes as described previously [33]. As seen in Figure 1e, the macrochromosomes are biarmed. C-banding revealed heterochromatin in the centromeres of several microchromosomes (Figure 1f). We also detected ITS in at least two chromosomal pairs (Figure 1g) and the rRNA probe produced a signal in one pair of microchromosomes (Figure 1h).

*Calumma brevicorne*: the karyotype is composed of 2n = 32 as described previously [34,37]. There are 18 biarmed macrochromosomes and 14 microchromosomes (Figure 1i). C-banding revealed heterochromatin in one arm of a middle-sized metacentric pair (Figure 1j). We detected ITS signals in several pairs of macrochromosomes (Figure 1k). FISH with rRNA probe showed a signal in pair 1 (Figure 1l). CGH did not detect any sexual differences (Figure 4a,b).

*Calumma* cf. *crypticum*: the karyotype is composed of 2n = 32 chromosomes. Eighteen chromosomes are biarmed macrochromosomes and 14 are microchromosomes (Figure 1m). In pair 9 we detected intraspecific polymorphism in the chromosome morphology. In four studied individuals both chromosomes were metacentric, in one male both were acrocentric and in one female one chromosome was metacentric and the other acrocentric (Figure 1m in box). C-banding showed heterochromatin in the centromeric/pericentromeric regions of several macrochromosomes and in one arm of a pair of middle-sized macrochromosomes (Figure 1n). On three pairs of macrochromosomes we detected ITS signals (Figure 1o). FISH with rRNA probe revealed a signal in pair 1 (Figure 1p). CGH did not uncover any sexual differences (Figure 4c,d).

*Calumma globifer*: the karyotype consists of 2n = 36 chromosomes as previously described [38]. Twelve chromosomes are biarmed macrochromosomes, 24 chromosomes can be assigned as microchromosomes (Figure 1q). C-banding revealed heterochromatin in the pericentromeric regions of four pairs of macrochromosomes (Figure 1r). No ITS signals were detected (Figure 1s). FISH with rRNA probe showed a signal in pair 2 (Figure 1t). CGH did not uncover any sexual differences (Figure 4e,f).

**Figure 2:**
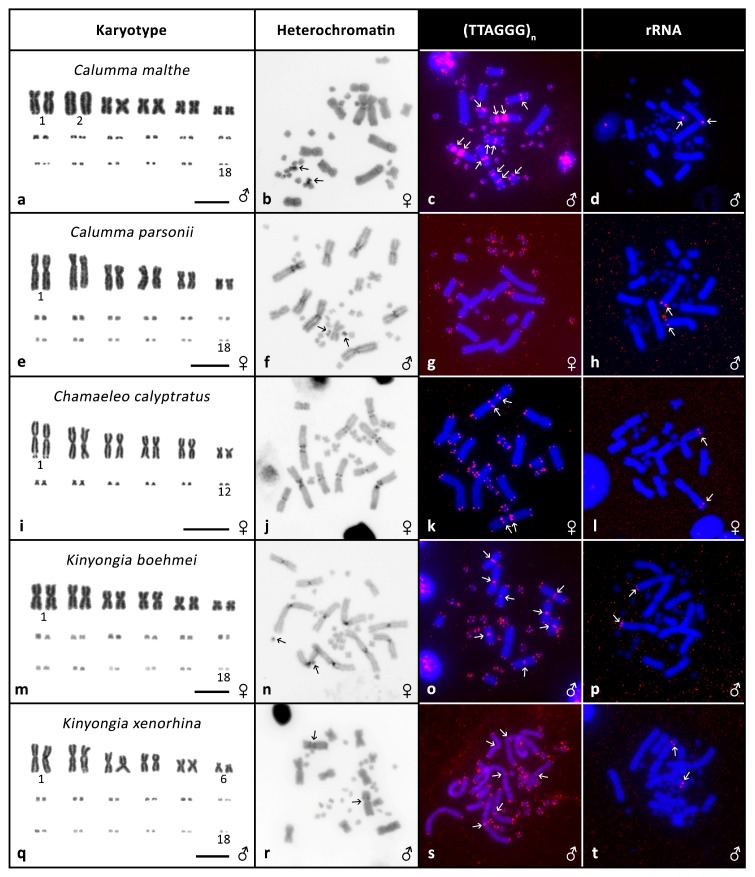
Cytogenetic data. Giemsa stained karyotypes (**a**,**e**,**i**,**m**,**q**), C-banding (**b**,**f**,**j**,**n**,**r**), FISH with telomeric probe (**c**,**g**,**k**,**o**,**s**) and FISH with rRNA probe (**d**,**h**,**l**,**p**,**t**) in *Calumma malthe*, *Calumma parsonii*, *Chamaeleo calyptratus*, *Kinyongia boehmei*, *Kinyongia xenorhina*. Arrows indicate signals.

*Calumma malthe*: the karyotype is composed of 2n = 36 chromosomes. There are 12 macro- and 24 microchromosomes. Pair 2 is acrocentric while all other macrochromosomes are biarmed (Figure 2a). C-banding revealed heterochromatin in a pair of microchromosomes (Figure 2b). ITS signals were detected in several macrochromosomes (Figure 2c) and rRNA probe produced a signal in pair 1 (Figure 2d).

*Calumma parsonii*: the karyotype is composed of 2n = 36 as previously described [37]. There are 12 biarmed macrochromosomes and 24 microchromosomes (Figure 2e). C-banding revealed heterochromatin in pericentromeric regions in four pairs of macrochromosomes and one microchromosome pair (Figure 2f). No ITS signals were detected (Figure 2g). FISH with rRNA probe showed a signal in pair 2 (Figure 2h). CGH did not uncover any sexual differences (Figure 4g,h).

*Chamaeleo calyptratus*: the karyotype is composed of 2n = 24 as previously described [39]. There are 12 biarmed macrochromosomes and 12 microchromosomes (Figure 2i). C-banding uncovered heterochromatin in the pericentomeric regions centromeres (Figure 2j). ITSs were present in a pair of macrochromosomes (Figure 2k). FISH with rRNA probe showed a signal in pair 1 (Figure 2l) and CGH did not reveal any sexual differences (Figure 4i,j).

*Kinyongia boehmei*: the karyotype is composed of 2n = 36 chromosomes of which 12 are biarmed macrochromosomes and 24 microchromosomes (Figure 2m). C-banding uncovered heterochromatin in the centromeres and on a pair of microchromosomes (Figure 2n). ITSs were present in several chromosomal pairs (Figure 2o). FISH with rRNA probe produced a signal in pair 2 (Figure 2p).

*Kinyongia xenorhina*: the karyotype consists of 2n = 36 of which 12 are biarmed macrochromosomes and 24 microchromosomes (Figure 2q). Pair 6 can also be classified as acrocentric as the p-arms are rather short. C-banding uncovered heterochromatin in the pericentromeric regions of one pair of macrochromosomes (Figure 2r). We detected ITS signals in several macrochromosomes (Figure 2s). FISH with rRNA probe showed a signal in pair 2 (Figure 2t).

**Figure 3:**
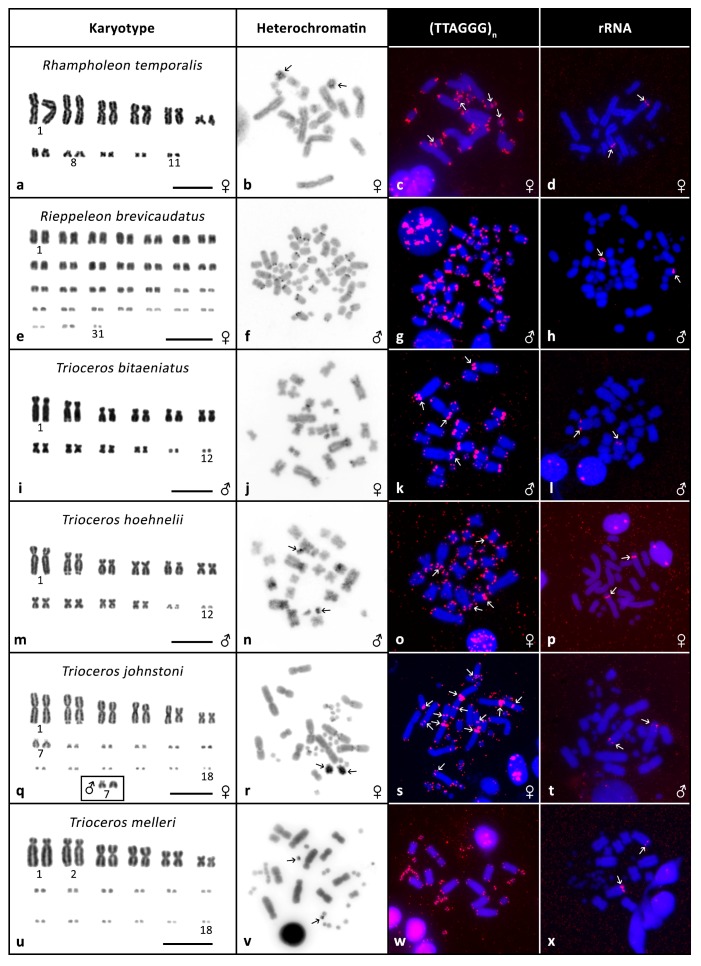
Cytogenetic data. Giemsa stained karyotypes (**a**,**e**,**i**,**m**,**q**,**u**), C-banding (**b**,**f**,**j**,**n**,**r**,**v**), FISH with telomeric probe (**c**,**g**,**k**,**o**,**s**,**w**) and FISH with rRNA probe (**d**,**h**,**l**,**p**,**t**,**x**) in *Rhampholeon temporalis*, *Rieppeleon brevicaudatus*, *Trioceros bitaeniatus*, *Trioceros hoehnelii*, *Trioceros johnstoni*, *Trioceros melleri*. Arrows indicate signals.

*Rhampholeon temporalis*: the karyotype consists of 2n = 22 where 10 chromosomes are biarmed macrochromosomes, six are intermediate-sized chromosomes and six are microchromosomes (Figure 3a). Heterochromatin accumulation was found in pair 8 of the intermediate-sized chromosomes (Figure 3b). C-banding revealed larger blocks of heterochromatin in one chromosome from this pair in the female and also a different position of its centromere. ITSs were found in the intermediate-sized chromosomes (Figure 3c). FISH with rRNA probe produced a signal in pair 2 (Figure 3d).

**Figure 4:**
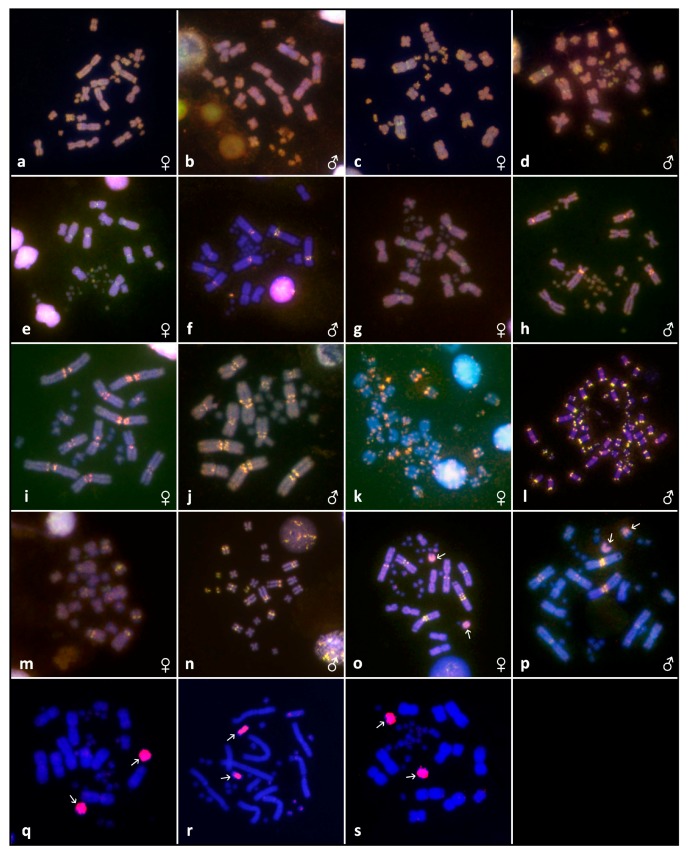
Results of comparative genomic hybridization (CGH) and chromosome painting. Results of CGH in female and male metaphases of *Calumma brevicorne* (**a**,**b**), *Calumma* cf. *crypticum* (**c**,**d**), *Calumma globifer* (**e**,**f**), *Calumma parsonii* (**g**,**h**), *Chamaeleo calyptratus* (**i**,**j**), *Rieppeleon brevicaudatus* (**k**,**l**), *Trioceros hoehnelii* (**m**,**n**) and *Trioceros johnstoni* (**o**,**p**). The male DNA is stained with fluorescein isothiocyanate (FITC; green colour), the female DNA with rhodamine (red colour). Regions common for genomes of both sexes are yellow (combination of green and red). Results of chromosome painting with microdissected chromosomes in *Trioceros johnstoni* (**q**–**s**). Chromosomes microdissected from pair 7 in female labelled with rhodamine hybridised separately in female metaphases (**q**,**r**). The probe prepared from male pair 7 also hybridized to both chromosomes of pair 7 in female metaphases (**s**).

*Rieppeleon brevicaudatus*: the karyotype consists of 2n = 62 (note the large number) and subsequently the chromosomes decline in size and are most likely all acrocentric (Figure 3e). C-banding uncovered heterochromatin in the centromeres/pericentromeric regions (Figure 3f). No ITS signals were detected, however in several chromosomes we observed a higher accumulation of telomeric sequences in the telomeric regions (Figure 3g). FISH with rRNA probe showed a signal in one pair, probably pair 6 or 7 (Figure 3h). CGH did not uncover any sexual differences (Figure 4k,l).

*Trioceros bitaeniatus*: the karyotype consists of 2n = 24 of which 20 are macrochromosomes and 4 microchromosomes as described previously [40]. All macrochromosomes are biarmed (Figure 3i). C-banding uncovered heterochromatin in the centromeres/pericentromeric regions (Figure 3j). ITSs were detected in pairs 1 and 2 (Figure 3k). In pair 2 we observed a secondary constriction at the site where the rRNA probe was bound (Figure 3l).

*Trioceros hoehnelii*: the karyotype is composed of 2n = 24 of which 20 are macrochromosomes and 4 microchromosomes as previously described [41]. All macrochromosomes are biarmed (Figure 3m). C-banding uncovered heterochromatin in the centromeres/pericentromeric regions and in a pair of microchromosomes (Figure 3n). We detected ITS accumulations in two pairs of macrochromosomes (Figure 3o). In pair 2 we observed a secondary constriction with the rRNA probe signal (Figure 3p). CGH did not uncover any sexual differences (Figure 4m,n).

*Trioceros johnstoni*: the karyotype is composed of 2n = 36 as described previously [33]. There are 14 macrochromosomes out of which 12 are biarmed and 22 microchromosomes (Figure 3q). In pair 7 we observed possible heteromorphy. In the female, it seems that the chromosomes in this pair differ in size while in the male they may differ in morphology (Figure 3q). C-banding revealed heterochromatin in the pericentromeric regions and also strongly in the only acrocentric macrochromosomes—pair 7, in both sexes (Figure 3r). We detected ITS signals in several chromosomal pairs (Figure 3s). FISH with rRNA probe produced a signal in pair 2 (Figure 3t). CGH revealed enrichment of female sequences in chromosome pair 7 in both sexes (Figure 4o,p). Chromosomes from pair 7 were microdissected and hybridised separately to female metaphases and the probes prepared from them displayed a signal in both chromosomes from each pair (Figure 4q,r). The probe prepared from male pair 7 also hybridized to both chromosomes of pair 7 in female metaphase (Figure 4s).

*Trioceros melleri*: the karyotype consists of 2n = 36 with 12 predominantly biarmed macrochromosomes and 24 microchromosomes (Figure 3u). C-banding revealed heterochromatin in one pair of microchromosome (Figure 3v). No ITS signals were detected (Figure 3w). In pair 2 we observed a secondary constriction where the rRNA probe produced a signal (Figure 3x).

### 3.3. The Reconstruction of Ancestral Chromosome Number

**Figure 5:**
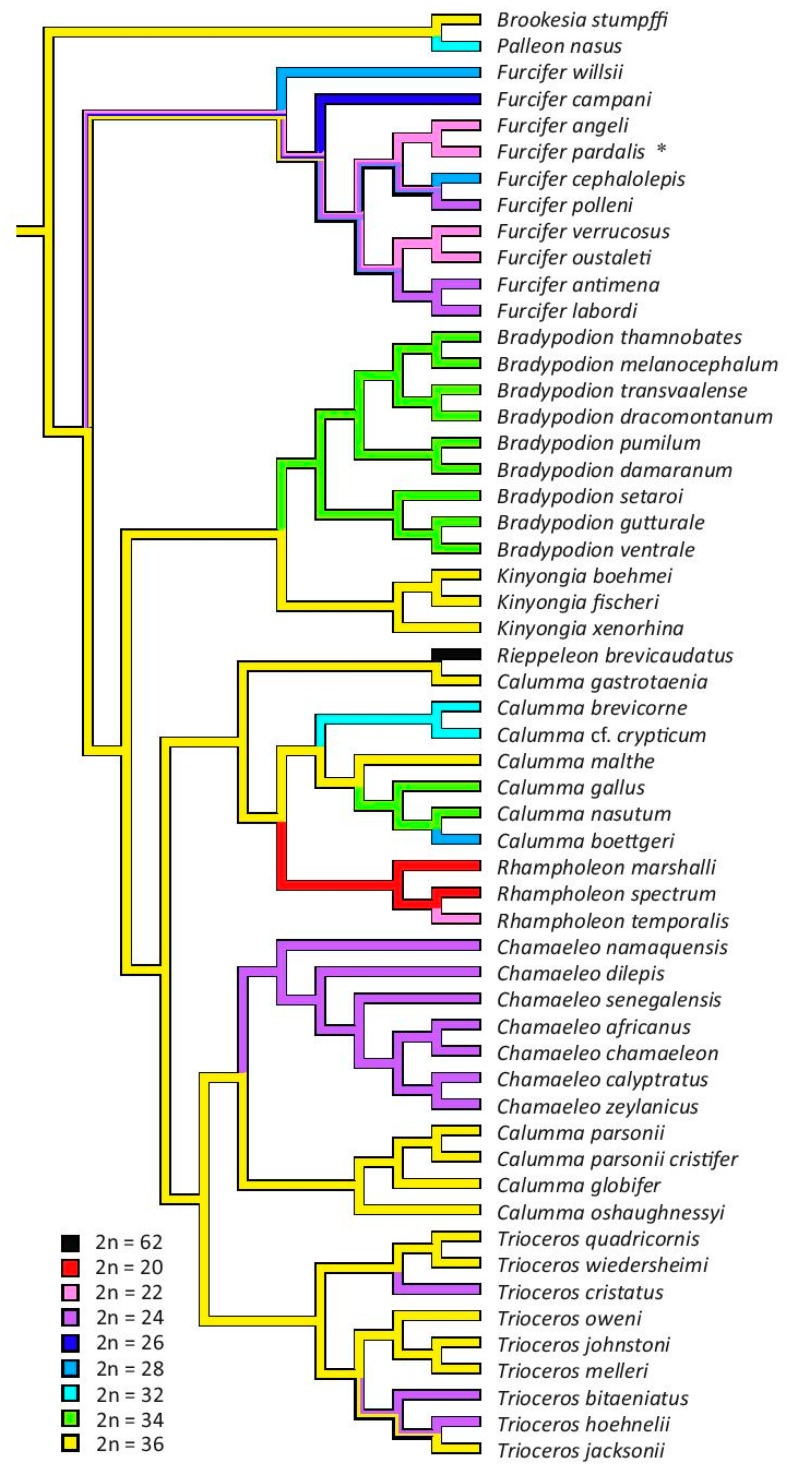
Phylogenetic analysis based on the tree by [32] with depicted number of chromosomes. Results of maximum parsimony analysis reconstructing ancestral chromosome number are visualized by colours of the branches. Asterisk (*) symbolises different chromosome number in female of *Furcifer pardalis* due to presence of neo-sex chromosomes.

**Figure 6:**
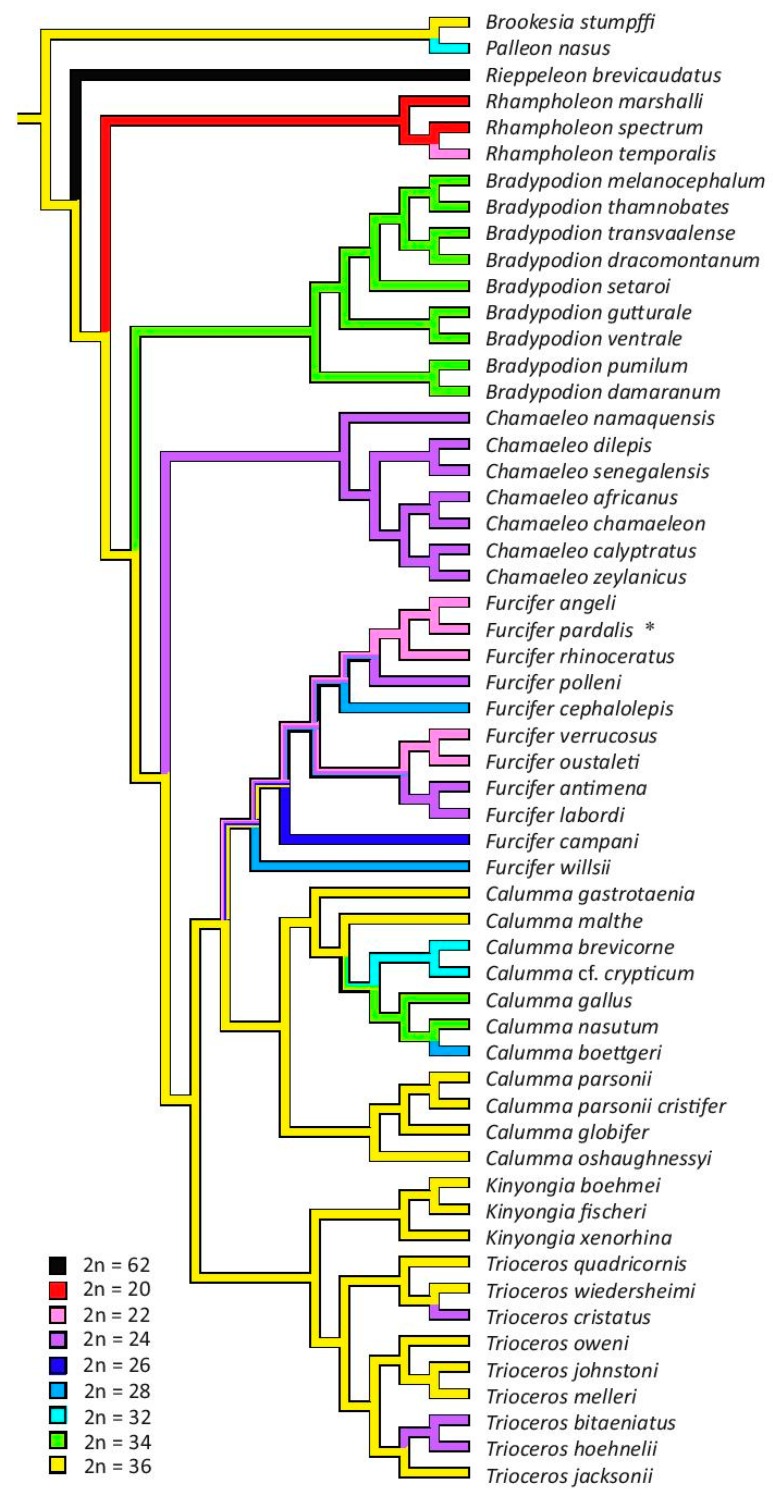
Phylogenetic analysis based on tree by [1] with depicted number of chromosomes. Results of maximum parsimony analysis reconstructing ancestral chromosome number are visualized by colours of the branches. Asterisk (*) symbolises different chromosome number in female of *Furcifer pardalis* due to presence of neo-sex chromosomes.

Maximum parsimony analysis reconstructed 2n = 36 as the ancestral chromosome number of the family Chamaeleonidae based on both alternative phylogenies (Figure 5 and Figure 6). The analyses based on both phylogenies predicted an ancestral state of 2n = 34 for the genus Bradypodion, 2n = 24 for the genus Chamaeleo, 2n = 20 for the genus Rhampholeon and 2n = 36 for the genera Kinyongia and Trioceros. In the phylogeny by [1], where the genus Calumma is monophyletic, the ancestral state for the genus was also reconstructed to 2n = 36. The ancestral chromosome number for the genus Furcifer remains unresolved in both trees (Figure 5 and Figure 6).

## 4. Discussion

In comparison to other squamate groups [3] chameleons have rather extensive variability in chromosome number and morphology. It was previously understood that chromosome numbers can vary from 2n = 20 to 2n = 36 in the family. However, we discovered that one species (*Rieppeleon brevicaudatus*) possesses a much higher chromosome number (2n = 62). The analyses of the ancestral chromosome number based on two contrasting phylogenies [1,32] are in agreement that the ancestral chromosome number for the whole family was most likely 2n = 36 (Figure 5 and Figure 6), but this conclusion is highly dependent on the assumption of homology of karyotype between *B. stumpffi*, members of the genera *Trioceros*, *Calumma* and *Kinyongia* with 2n = 36 chromosomes. The position of the rDNA genes differs between *B. stumpffi*, where the rRNA probe hybridized with a microchromosomal pair, and the other three genera, where rRNA genes are located in a large macrochromosome, which suggests that the karyotypes with 2n = 36 are not homologous. Nevertheless, the position of rRNA genes is highly evolutionary labile in some lizard groups [16] and it is therefore not so informative. The ancestral karyotype with 2n = 36 chromosomes has also been suggested for outgroup lineages of chameleons, such as dragon lizards (Agamidae) and iguanas [10,42,43]. Further research based on chromosome painting, gene mapping and other techniques enabling testing of chromosome homology is needed to determine the ancestral karyotype in chameleons and its homology to the reconstructed ancestral karyotype to the whole group Iguania. Our phylogenetic analysis shows that there is a tendency for chromosome number reduction within chameleons and that the evolution of genome organisation probably followed pathways involving chromosome rearrangement leading to lower chromosome numbers (e.g., Robertsonian and tandem fusions).

On the other hand, the significant increase in chromosome number in *Rieppeleon brevicaudatus* is clearly derived (Figure 5 and Figure 6). Here, the morphology of the chromosomes also differs extensively. In most species, the basic genome organisation stays very similar despite the variation in chromosome number. Usually there are 10 to 20 macrochromosomes in the karyotype with the remainder represented by microchromosomes. In macrochromosomes, the majority of chromosomes are biarmed. Therefore, the karyotype of *R. brevicaudatus* with 62 acrocentric chromosomes with gradually decreasing size is really exceptional and many chromosome splits must have taken place during its formation. As all chromosomes are acrocentric we presume that karyotypes such as this one can originate via Robertsonian fissions of biarmed chromosomes. The genome organisation of *R. brevicaudatus* is unusual not only within chameleons but even among all squamate reptiles. Karyotypes of such a high diploid chromosome number occur very rarely in squamates and 2n = 62 is most likely the highest diploid chromosome number within the whole order [3]. The same chromosome number has been reported only in one other squamate; the gymnophtalmid *Notobrachia ablephara* [44]. 

In our analyses, we also discovered rather extensive variability in the presence of ITSs, with the number and position differing between the twelve species in which we detected them (see Figure 1, Figure 2 and Figure 3 for examples). We were not able to detect any stronger phylogenetic signal in the presence or absence of the ITSs, as even relatively closely related species such as *T. bitaeniatus* and *T. hoehnelii* differ in the occurrence of ITS. Recently we documented that the presence of ITSs in squamate reptiles is more common than previously expected and that the variability of their presence is in general remarkable [27]. In chameleons, it seems that ITS may not often be a remnant of interchromosomal rearrangements as species with the same chromosome number and similar chromosome morphology differ in ITS occurrence.

In contrast to chromosome number and ITS variability, we observed only a very limited variability in the position of rDNA accumulations (nucleolar organising regions, NORs) between species. In the majority of species, the rDNA signal was detected on the second largest pair of chromosomes. It suggests that despite the variability in chromosome number, certain chromosome content may be rather stable and larger syntenic blocks may have remained unchanged throughout the evolution of chameleons as we previously documented on a wider scale of reptile phylogeny [39,45]. Such stability in the position of rDNA is not typical for many animal lineages [16,46] where the genes are present on very different chromosomes in different species.

In our study, we also focused on searching for differentiated sex chromosomes, which we previously detected in two species of the genus *Furcifer* [7]. However, in the 12 chameleon species where we could examine both sexes, we did not find clearly differentiated sex chromosomes. In most species, we have not detected any differences in the karyotype of males and females using either C-banding or CGH. The stable sex ratios across temperatures in chameleons suggests that their sex chromosomes are most likely at the early stage of differentiation and highly differentiated sex chromosomes within the genus *Furcifer* represent a derived state. The only species where we may have detected evidence for sexual differences in karyotype are *Rhampholeon temporalis* and *Trioceros johnstoni*. In *R. temporalis*, where we had only female samples available, there was one pair which showed an accumulation of heterochromatin and the chromosomes may be heteromorphic (Figure 3b). Similarly, in *T. johnstoni* we observed heteromorphism in one chromosomal pair (pair 7; Figure 3r). CGH suggested that this chromosome pair might be enriched in female-specific sequences (Figure 4o,p); however, we cannot rule out that this signal is not driven by non-sex specific accumulations of repetitive elements in the female specimen whose DNA we used for CGH. In the FISH with the microdissected chromosomes both probes hybridised with both chromosomes from the pair (Figure 4q–s), which suggests that these peculiar chromosomes of *T. johnstoni* are either autosomes with variable accumulations of repetitive sequences, or sequentially almost undifferentiated sex chromosomes. CGH, as well as chromosome painting technique, are not sensitive enough to visualise differences among chromosomes restricted to a small region [16]. Additional studies including both cytogenetic and genomic approaches are needed to clarify the nature of these chromosomes.

## Supplementary Materials

The following are available online at www.mdpi.com/2073-4425/8/12/382/s1. Table S1: Number of individuals, accession numbers of COI haplotypes, BLAST similarity and methods used for all studied species.
